# Respiratory Viral Infection Alters the Gut Microbiota by Inducing Inappetence

**DOI:** 10.1128/mBio.03236-19

**Published:** 2020-02-18

**Authors:** Helen T. Groves, Sophie L. Higham, Miriam F. Moffatt, Michael J. Cox, John S. Tregoning

**Affiliations:** a Mucosal Infection and Immunity Group, Section of Virology, Department of Medicine, St Mary’s Campus, Imperial College London, London, United Kingdom; b National Heart & Lung Institute, Imperial College London, London, United Kingdom; c Respiratory Biomedical Research Unit, Royal Brompton & Harefield NHS Trust, London, United Kingdom; University of Pittsburgh School of Medicine

**Keywords:** appetite, metabolome, microbiome, T cell, lung infection, respiratory syncytial virus

## Abstract

Respiratory viral infections are extremely common, but their impacts on the composition and function of the gut microbiota are poorly understood. We previously observed a significant change in the gut microbiota after viral lung infection. Here, we show that weight loss during respiratory syncytial virus (RSV) or influenza virus infection was due to decreased food consumption, and that the fasting of mice altered gut microbiota composition independently of infection. While the acute phase tumor necrosis factor alpha (TNF-α) response drove early weight loss and inappetence during RSV infection, this was not sufficient to induce changes in the gut microbiota. However, the depletion of CD8^+^ cells increased food intake and prevented weight loss, resulting in a reversal of the gut microbiota changes normally observed during RSV infection. Viral infection also led to changes in the fecal gut metabolome, with a significant shift in lipid metabolism. Sphingolipids, polyunsaturated fatty acids (PUFAs), and the short-chain fatty acid (SCFA) valerate were all increased in abundance in the fecal metabolome following RSV infection. Whether this and the impact of infection-induced anorexia on the gut microbiota are part of a protective anti-inflammatory response during respiratory viral infections remains to be determined.

## Abstract (precis)

The gut microbiota has an important role in health and disease: gut bacteria can generate metabolites that alter the function of immune cells systemically. Understanding the factors that can lead to changes in the gut microbiome may help to inform therapeutic interventions. This is the first study to systematically dissect the pathway of events from viral lung infection to changes in gut microbiota. We show that the cellular immune response to viral lung infection induces inappetence, which in turn alters the gut microbiome and metabolome. Strikingly, there was an increase in lipids that have been associated with the resolution of disease. This opens up new paths of investigation: first, what is the (presumably secreted) factor made by the T cells that can induce inappetence? Second, is inappetence an adaptation that accelerates recovery from infection, and if so, does the microbiome play a role in this?

## INTRODUCTION

The gut microbiota plays many critical roles in maintaining human health. These include local effects such as metabolizing nondigestible nutrients, providing colonization resistance against gut infection, helping maintain intestinal barrier function, and educating the immune system (1). It is increasingly appreciated that, in addition to its local effects, the gut microbiota also has systemic effects on health, for example, through the production of anti-inflammatory metabolites such as short-chain fatty acids (SCFAs) (2). In the context of respiratory disease, most studies have focused on how the gut microbiota influences immune responses in the airways (3). Changes in gut microbiota composition can change the gut metabolome, with a subsequent impact on host immune function (4). Mice fed a diet high in fermentable fiber have decreased lung damage and increased survival during influenza virus infection due to an increase in fecal and serum SCFA (5). Likewise, increased abundance of the polyunsaturated fatty acid (PUFA) docosahexaenoic acid (DHA) after *Lactobacillus* supplementation led to reduced lung inflammation and damage during respiratory syncytial virus (RSV) infection in mice (6).

However, several studies have demonstrated that respiratory infections are associated with a change in the composition of the gut microbiota (7–12). We previously observed that viral lung infections alter the gut microbiota, leading to an increase in the relative abundance of *Bacteroidetes* and a decrease in the relative abundance of *Firmicutes* (10). In our studies, we did not identify a mechanism that linked viral lung infection with changes in the gut microbiota. We did note that changes in overall gut microbiota composition after either RSV or influenza A virus infection were similar. This implies that the underlying mechanism is common to both infections and therefore not a pathogen-specific immune effect as suggested elsewhere (7, 8). In mice, one common symptom after RSV or influenza infection is weight loss, part of a wider pattern of sickness behaviors (13). This weight loss has been associated with reduced food intake after influenza infection in mice (14, 15). Though the effect of RSV infection on food intake in mice has not been published, mild anorexia has been observed in RSV-infected preterm lambs (16). Loss of appetite is also reported after human influenza virus and RSV infection (17, 18). Reduced calorie intake in humans and mice has been associated with a significant increase in the ratio of *Bacteroidetes* to *Firmicutes* abundance (19–21), similar to that observed in our prior viral infection study (10). This suggests that changes in the gut microbiota seen after respiratory viral infection might be driven by reduced food intake.

Therefore, the aim of this study was to understand the interplay between infection, food intake, the gut microbiome, and the gut metabolome. We show that changes in the gut microbiome after infection are caused by reduced food intake and that this is associated with CD8^+^ T cells. Additionally, we observed a significant change in the gut metabolome after lung infection, with a significant increase in the levels of lipids produced.

## RESULTS

### Respiratory infection reduces food consumption and alters the gut microbiota.

**FIG 1:**
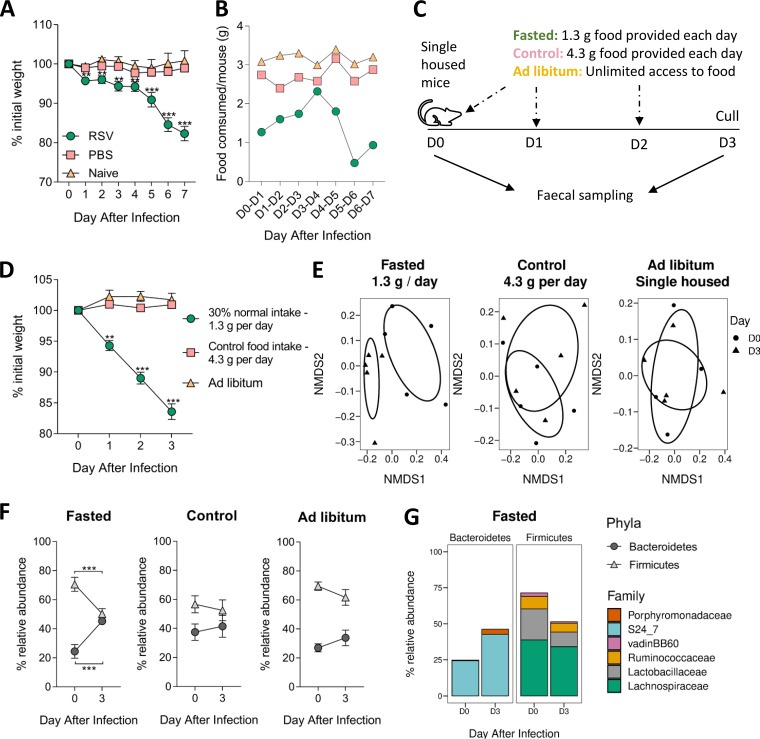
Reduced food consumption alters the composition of the gut microbiota increases the relative abundance of *Bacteroidetes* and decreases *Firmicutes*. Mice were intranasally infected with 2 × 10^6^ PFU/ml RSV-A2, intranasally dosed with sterile PBS, or left naive. The weights of individual mice (A) and food consumption of the entire cage (B) were measured every day. Fasted mice were provided with 1.3 g food per day per mouse, which was 30% of control food intake (4.3 g per mouse per day). (C) Fecal samples were taken from fasted and control mice and from mice with unlimited access to food (*ad libitum)* at day 0 and day 3. (D) Weight loss in fasted mice. (E) Gut microbiota diversity composition compared to that prefasting (*P* = 0.01). (F) Relative abundances of *Bacteroidetes* and *Firmicutes* after infection. (G) Family level analysis of gut microbiota response after infection. *n* = 5 mice per group. These data are representative of 2 studies. Weight loss and changes in phyla/family abundance tested using repeated measures two-way ANOVA, with Dunnett’s and Sidak’s multiple-comparison tests, respectively. Change in beta diversity was visualized using NMDS on Brays-Curtis dissimilarity matrix, using ADONIS to test for significant difference. **, *P ≤ *0.01; ***, *P ≤ *0.001.

We first investigated the link between weight loss and food intake after infection. Mice infected with RSV lost weight from the first day after infection, stabilizing between days 1 and 4 and then losing more weight from day 5, resulting in approximately 15% to 20% weight loss by day 7 (Fig. 1A). There was no weight loss in intranasally phosphate-buffered saline (PBS)-dosed or naive mice. Food consumption mirrored weight loss. The average amount of food consumed by one uninfected mouse was 3 g (±0.3 g) per day. RSV-infected mice immediately ate less following infection (day 0 [D0] to D1, 1.3 g). Food consumption in RSV-infected mice increased between days 1 and 4. After day 4, RSV-infected mice ate less food each day, with the nadir average food consumption of 0.5 g per mouse on day 6 (Fig. 1B). We saw a similar effect after influenza infection (see Fig. S1 in the supplemental material). This strongly suggests that respiratory viral infection induces inappetence, which then reduces food consumption leading to weight loss.

Having observed that reduced food intake during infection was associated with weight loss, we wanted to investigate whether there was a link between food intake, weight loss, and changes in gut microbiota outside the context of infection. To do this, we restricted access to food to reflect the weight loss seen after infection (Fig. 1C). Mice were singly housed to control food intake per mouse, but as mice are social animals, the stress of individual housing could impact the gut microbiota. To control for this, a cage of single-housed mice with *ad libitum* food access was monitored alongside cohoused animals with *ad libitum* food access to account for any single-housing stress-induced changes. No differences in food consumption or gut microbiota diversity were seen between individually housed or cohoused mice with *ad libitum* food access (see Fig. S2). Fasted mice (with access to 1.3 g food per day) lost approximately 15% total weight by day 3 (Fig. 1D). Neither the control mice receiving 4.3 g food per day nor the singly housed mice with *ad libitum* access to food lost weight.

Feces were collected at day 0 and 3 to sample the gut microbiota. Beta diversity was significantly different following fasting, indicating a shift in overall gut microbiota composition (*P* < 0.01) (Fig. 1E). There was no difference in beta diversity between days 0 and 3 in control mice or singly housed mice with *ad libitum* food access. At the phylum level, the gut microbiota of fasted mice had a significantly higher relative abundance of *Bacteroidetes* and a significantly lower relative abundance of *Firmicutes* than at baseline (Fig. 1F). There was no change in these phyla in control or singly housed *ad libitum*-fed mice. At the family level, the major change seen was an increase in the abundance of the S24_7 family (also known as *Muribaculaceae* [22]) (Fig. 1G). This pattern was very similar to that previously observed after RSV infection (10).

### TNF-α is associated with weight loss after infection but not with changes in the gut microbiota.

Having observed that viral infection reduces food intake and a reduction in food intake alters the gut microbiota, we wanted to determine the role of host immune factors in inappetence after respiratory viral infection. One potential factor is the proinflammatory cytokine tumor necrosis factor alpha (TNF-α). Intraperitoneal injection of recombinant murine TNF-α (r-TNF-α) was previously shown to induce weight loss in mice (23). TNF-α is elevated in the airways in response to RSV infection in both humans and mice (24, 25), and blocking TNF-α during RSV infection has been shown to reduce weight loss (26, 27). Therefore, we examined the roles of TNF-α in inappetence, food intake, and the gut microbiota after RSV infection.

**FIG 2:**
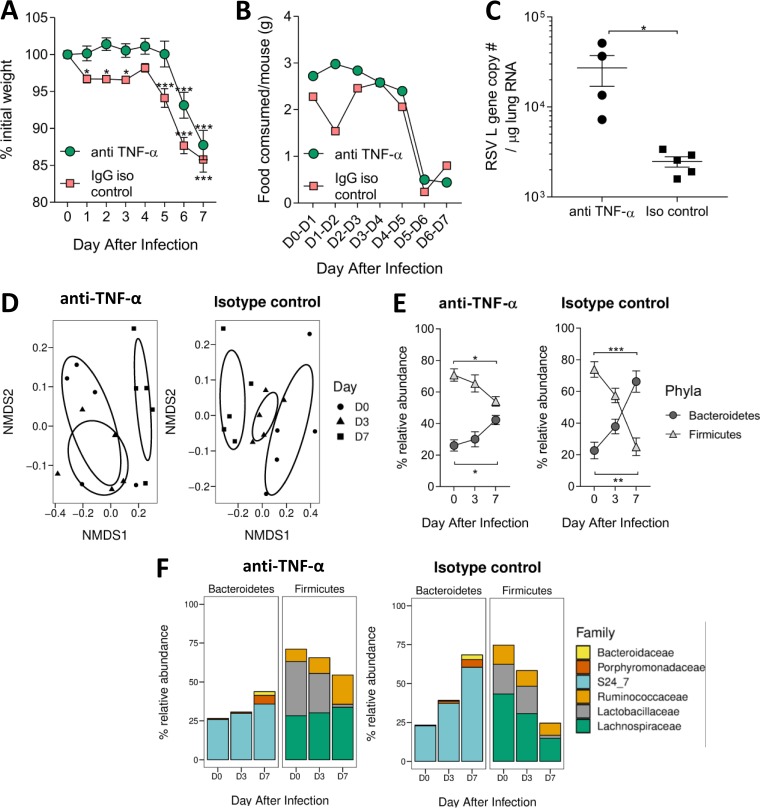
TNF-α may drive early weight loss observed during RSV infection; however, blocking TNF-α does not completely prevent weight loss. Mice were i.p. injected with 450 μg/ml anti-TNF-α antibody or isotype control before (−4 h) and after (day 2 [D2], D4, and D6) RSV infection. Fecal samples were taken before infection (D0) and at days 3 and 7. Weight loss (A) and food consumption (B) were measured after infection. (C) RSV viral load in the lungs was quantified using an RSV L gene qPCR. (D) Gut microbiota diversity was measured at day 3 and 7 after infection. (E) Relative abundances of *Bacteroidetes* and *Firmicutes* phyla after infection. (F) Family level analysis of gut microbiota response after infection. *n* = 5 mice per group. Results representative of two experiments. *, *P ≤ *0.05; **, *P ≤ *0.01; ***, *P ≤ *0.001.

Mice were injected intraperitoneally with an anti-TNF-α monoclonal or a control antibody on days 0, 2, 4, and 6 of infection (27), with fecal samples taken on days 0, 3, and 7 of infection. Anti-TNF-α-treated mice did not lose any weight during the first 5 days of RSV infection, in contrast to the isotype control group, which lost weight immediately following infection. However, after 5 days, anti-TNF-α-treated mice lost weight rapidly, and by day 7, there was no difference in weight loss between them and the control group (Fig. 2A). Infected isotype control mice reduced their average food intake between days 1 and 2 of infection (Fig. 2B), whereas blocking TNF-α prevented inappetence during the early stages of infection (days 1 to 4). From day 5 onwards, TNF-α-blocked mice had reduced food intake. Anti-TNF-α-treated mice had a significantly higher RSV lung viral load at day 7 compared to that in the isotype control group (Fig. 2C). There was no effect of TNF-α blockade on the T cell response (see Fig. S3). Beta diversity of the microbiota corresponded with weight loss during RSV infection. RSV-infected isotype control mice experienced a significant shift in gut microbiota beta diversity at both days 3 and 7, while TNF-α-depleted mice only had a significantly altered gut microbiota beta diversity at day 7 (Fig. 2D). At day 7, the change in the relative abundance of *Bacteroidetes* and *Firmicutes* during RSV infection was reduced in anti-TNF-α-treated mice compared to that in the control group (Fig. 2E). Anti-TNF-α-treated mice had significantly less *Ruminococcaceae* and *Lactobacillaceae* in their gut microbiota at day 7 than at baseline, whereas isotype control mice had fewer *Lachnospiraceae* and more S24_7 (Fig. 2F).

**FIG 3:**
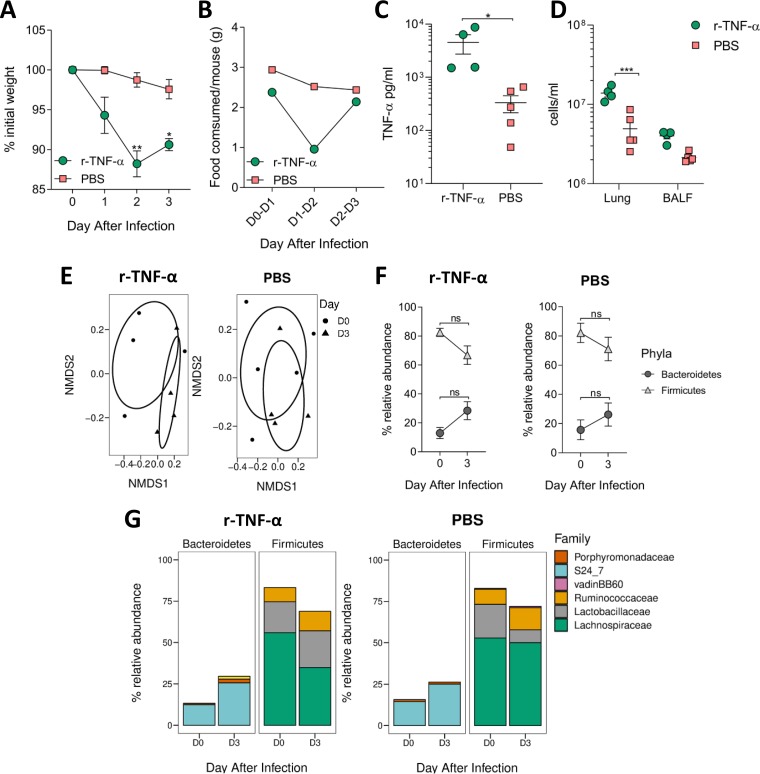
Acutely increasing airway TNF-α drives reduced food consumption, weight loss, and lung cell infiltration but is not sufficient to alter the gut microbiota composition. Mice were dosed intranasally with 3 μg recombinant TNF-α or PBS every day for 3 days. Fecal samples were taken before (day 0) and after (day 3). Weight loss (A) and food consumption (B) were measured during intranasal r-TNF-α dosing. Airway TNF-α levels (C) and total number of cells in the lungs (D) after intranasal r-TNF-α dosing. (E) Gut microbiota diversity after r-TNF-α or PBS dosing. Relative abundances of *Bacteroidetes* or *Firmicutes* (F) and bacterial families (G) after dosing. *n* = 4 to 5 mice per group. Results are representative of two experiments. Weight loss and changes in phyla/family abundance tested using repeated measures two-way ANOVA, with Dunnet’s and Sidak’s multiple-comparison tests, respectively. Change in diversity was visualized using NMDS on Brays-Curtis dissimilarity matrix, using ADONIS to test for significant difference. Differences in cytokine levels/cell numbers/percentages were tested using unpaired *t* tests. *, *P ≤ *0.05; **, *P ≤ *0.01; ***, *P ≤ *0.001.

Since we saw a smaller change in gut microbiota in anti-TNF-α-treated mice following RSV infection, we investigated whether increasing airway TNF-α could alter the gut microbiota in the absence of infection. Based on the doses and subsequent weight loss reported after intraperitoneal r-TNF-α administration by Biesmans et al. (23), we intranasally delivered 3 μg r-TNF-α per mouse, aiming for 1 g weight loss every 24 h. Mice intranasally dosed with r-TNF-α began losing weight on day 1, with peak weight loss on day 2. Mice began to recover slightly by day 3, although there was still significant weight loss compared to the baseline (*P* < 0.05) (Fig. 3A). Food intake mirrored weight loss: r-TNF-α-dosed mice ate less between 1 and 2 days after infection (Fig. 3B). They began to eat more on day 3 despite receiving another dose of r-TNF-α. TNF-α levels in the airways of r-TNF-α-dosed mice were significantly higher than in PBS-treated mice (*P* < 0.05, Fig. 3C).

r-TNF-α-dosed mice had significantly higher total lung cell counts than PBS-dosed mice (*P* < 0.001) (Fig. 3D). However, while it induced acute weight loss, increasing the levels of airway TNF-α had no effect on gut microbiota beta diversity (Fig. 3E). Both r-TNF-α- and PBS-dosed mice showed a trend toward increased relative abundance of *Bacteroidetes* and decreased relative abundance of *Firmicutes* (Fig. 3F). There was no significant change in the abundance of families belonging to either phyla (Fig. 3G). These data indicate that TNF-α is not the sole driver behind inappetence and weight loss during RSV infection.

### Depleting CD8^+^ T cells during RSV infection reduces inappetence and reverses changes in the gut microbiota.

Blocking CD8^+^ T cells during RSV infection was shown previously to reduce weight loss and increase viral load (28, 29), while adoptive transfer of RSV-specific CD8^+^ T cells into naive mice prior to RSV infection increases weight loss (30). We therefore investigated whether CD8^+^ T cells have roles in inappetence and the changes observed in the gut microbiota after infection.

**FIG 4:**
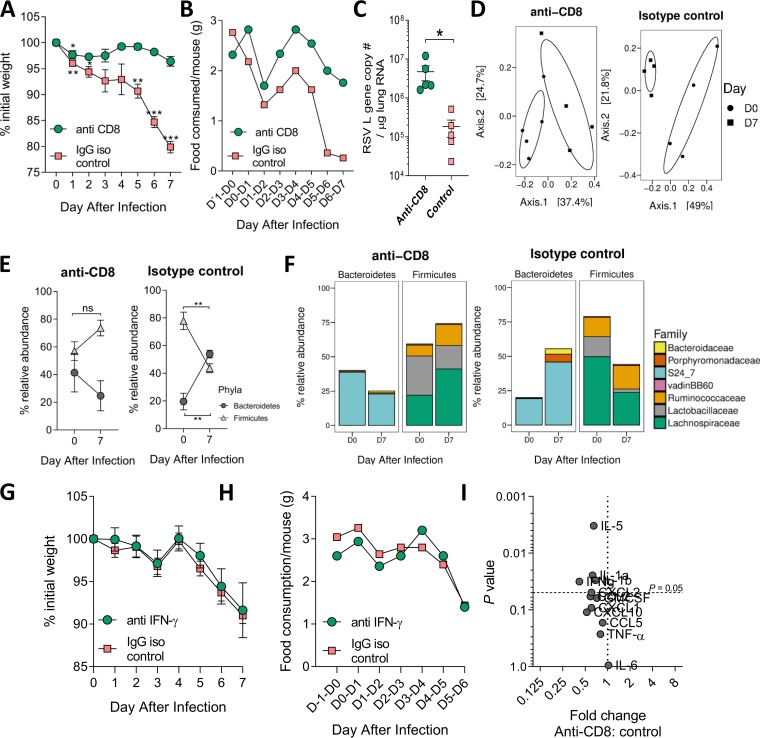
Depletion of CD8^+^ T cells during RSV infection prevents weight loss and associated gut microbiota changes, but this is not due to decreased IFN-γ production. CD8^+^ T cells were depleted during RSV infection by using mouse anti-CD8 monoclonal antibody. Mice were i.p. injected with 500 μg/ml antibody or isotype control before (day −1) and after (D2 and D5) RSV infection. Fecal samples were taken at D0 and D7. Weight loss (A) and food consumption (B) were measured after infection. (C) RSV L gene lung viral load was measured after infection at day 7. (D) Gut microbiota diversity measured at D0 and D7. (E) Relative abundances of *Bacteroidetes* and *Firmicutes* after infection (F). Family-level analysis of response after infection. IFN-γ was blocked by injecting mice i.p. with 500 μg/ml anti-mouse IFN-γ antibody or isotype control before (−4 h) and after (D2, D4, and D6) RSV infection. Effect of IFN-γ depletion on weight loss (G) and food consumption (H) during RSV infection. (I) Fold change in lung cytokine levels following CD8 depletion at day 7 compared to that in isotype control group. Significance for weight loss and phyla/family abundance tested using two-way RM ANOVA with Dunnet’s and Sidak’s multiple-comparison tests, respectively. Beta diversity was visualized using principal-coordinate analysis (PCoA) on a Brays-Curtis dissimilarity matrix, as there were insufficient data to perform NMDS. *n* = 4 to 5 mice per group. Results are from a single experiment.

CD8^+^ cells were depleted by systemic injection of an anti-CD8^+^ monoclonal antibody on days −1, 2, and 5 of infection. Control mice were injected at the same time with an IgG isotype control antibody. There was no effect on early weight loss; however, the depletion of CD8^+^ cells prevented all subsequent weight loss compared to that in control mice (*P* < 0.01) (Fig. 4A). The depletion of CD8^+^ cells reduced, but did not completely prevent, inappetence (Fig. 4B): anti-CD8-treated mice only ate an average of 1.8 to 2 g of food per day over the last 2 days of infection compared to the usual 3 g/mouse/day. This was higher than the infected control group, which consumed an average of 0.3 to 0.5 g food per mouse on days 6 and 7. Viral load in the lungs significantly increased following CD8^+^ cell depletion (Fig. 4C). CD8^+^ depletion significantly reduced the numbers of CD8^+^ T cells in the lungs after infection but not the overall number of cells recruited to the airways (see Fig. S4).

Despite anti-CD8 treatment preventing weight loss during infection, there was a significant shift in gut microbiota beta diversity from days 0 to 7 (Fig. 4D). Interestingly, unlike in the control group, there was no change in the relative abundance of *Bacteroidetes* or *Firmicutes* following CD8^+^ cell depletion (Fig. 4E). The blockade of CD8^+^ cells reversed the effect of RSV infection on the bacterial families detected in the gut, with a significant decrease in the relative abundance of S24_7 and an increase in *Lachnospiraceae* (Fig. 4F). Since gamma interferon (IFN-γ) is associated with CD8, we investigated whether IFN-γ blockade would have an impact (see Fig. S5). There was no effect of IFN-γ blockade on weight loss (Fig. 4G) or food intake (Fig. 4H) after RSV infection. To identify possible mediators that might have an impact on weight loss after RSV infection for future studies, we measured lung cytokines by Luminex after CD8 depletion. There was a significant decrease in lung interleukin (IL)-5, IL-1α, and IL-1β after CD8 depletion (Fig. 4I). The depletion of CD8^+^ cells during RSV infection reduced inappetence and immediate weight loss, with a marked effect on the gut microbiota.

10.1128/mBio.03236-19.1 FIG S1 Influenza virus infection reduced food consumption and resulted in weight loss. Mice were infected intranasally with 4 × 10^5^ PFU A/Eng/195/09 influenza virus, dosed with sterile PBS, or left naive. Mice were weighed individually (A) and food consumption of the entire cage (B) was measured every day postinfection. *n* = 5 mice per group/cage. Download FIG S1, TIF file, 0.8 MB. Copyright © 2020 Groves et al. 2020 Groves et al. This content is distributed under the terms of the Creative Commons Attribution 4.0 International license.

10.1128/mBio.03236-19.2 FIG S2 Individually housing mice does not alter their food intake or gut microbiota profiles. Mice were either individually housed for 10 days or cohoused in groups of five. *Ad libitum* food intake was measured over three days (A), with fecal samples taken at day 0 and day 3 for diversity analysis (B). Download FIG S2, TIF file, 1.1 MB. Copyright © 2020 Groves et al. 2020 Groves et al. This content is distributed under the terms of the Creative Commons Attribution 4.0 International license.

10.1128/mBio.03236-19.3 FIG S3 Characterization of anti-TNF-α treatment during RSV infection. Mice were i.p. injected with 450 μg anti-TNF-α antibody or isotype control before (−4 h) and after (D2, D4, and D6) RSV infection. (A) TNF-α levels were measured in the BAL fluid by ELISA (B). Luminex was used to measure fold change of additional cytokines in the BAL fluid. (C) Total lung and BAL fluid cell counts. Percentages of CD3^+^ lymphocytes (D), CD4^+^ T cells (E), and CD8^+^ T cells (F) in the lungs following TNF-α blockage compared to those in mice receiving isotype control. Unpaired *t* test. **, *P* ≤ 0.01. Download FIG S3, TIF file, 1.3 MB. Copyright © 2020 Groves et al. 2020 Groves et al. This content is distributed under the terms of the Creative Commons Attribution 4.0 International license.

10.1128/mBio.03236-19.4 FIG S4 Characterization of CD8^+^ T-cell depletion during RSV infection. (A) There was no difference in total lung or airway (BAL fluid) cell numbers after depleting CD8 ^+^ T cells during RSV infection. However, there were significantly fewer CD3^+^ lymphocytes in the lungs of anti-CD8 treated mice (B), and proportionally, there was a much greater percentage of CD4 ^+^ T cells (C) and significantly fewer CD8 ^+^ T cells (D) in the lungs. Significance was detected using either two-way ANOVA or unpaired *t* test. ***, *P* ≤ 0.001. Download FIG S4, TIF file, 0.9 MB. Copyright © 2020 Groves et al. 2020 Groves et al. This content is distributed under the terms of the Creative Commons Attribution 4.0 International license.

10.1128/mBio.03236-19.5 FIG S5 Characterization of anti-IFN-γ treatment during RSV infection. Mice were i.p. injected with 500 μg anti-IFN-γ antibody or isotype control before (−4 h) and after (D2, D4, and D6) RSV infection. IFN-γ levels were measured in the lungs (A) or BAL fluid (B) by ELISA. Total number of cells in the BAL fluid (C), percentages of CD3^+^ lymphocytes (D), CD8^+^ T cells (E), or CD4^+^ T cells (F) in the lungs of IFN-γ blocked mice compared to those in the isotype control. Unpaired *t* test. *, *P* ≤ 0.05. Download FIG S5, TIF file, 1.0 MB. Copyright © 2020 Groves et al. 2020 Groves et al. This content is distributed under the terms of the Creative Commons Attribution 4.0 International license.

### RSV infection changes the fecal metabolome, altering lipid metabolism.

We hypothesized that reduced food intake during infection would decrease overall nutritional availability within the gut, impacting host and microbiota metabolism and metabolite levels. The fecal metabolome has been shown to be altered by both acute and long-term fasting (31–33). This is likely to be a combination of direct effects of fasting on host metabolism and indirect effects of altered gut microbiota composition and metabolism.

**FIG 5:**
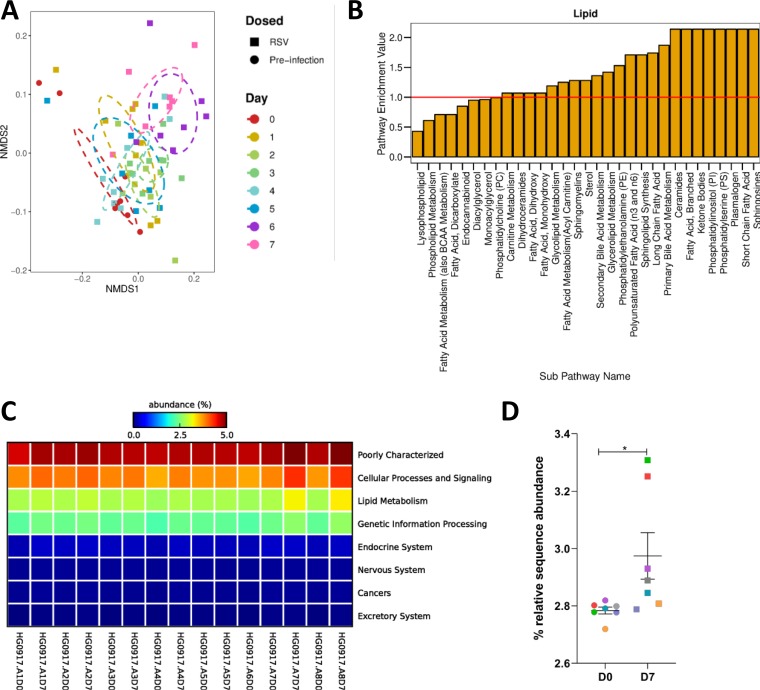
The fecal metabolome, and particularly, lipid metabolism, is altered during RSV infection, with an associated change in the predicted functional capacity of the gut microbiota. Mice were infected with RSV, and fecal samples were taken before infection (D0) and at every day postinfection until day 7 (D7). The fecal metabolome was profiled using HPLC-MS. (A) The change in the overall composition of the fecal metabolome over time during RSV infection, visualized using NMDS on a Bray-Curtis dissimilarity matrix. (B) Pathway enrichment analysis examining metabolic subpathways (*x* axis) belonging to the lipid metabolism superpathway, which contain significantly altered abundances of metabolites after RSV infection (day 7) compared to that before infection. A pathway enrichment value of >1 (red line) indicates that this pathway contains more experimentally different metabolites relative to the study as a whole. (C) 16S rRNA gene sequencing data corresponding to the metabolome samples were analyzed using PICRUSt to predict changes in the microbiota metagenome following RSV infection. Predicted KEGG orthologs (KOs) were collapsed into KEGG pathways. Seven known KEGG pathways were found to contain significantly altered abundances of predicted KOs, comparing D0 samples to D7 samples. (D) Relative sequence abundances of predicted KOs classified as belonging to lipid metabolism pathways (colors correspond to matching samples). *, *P* ≤ 0.05. *n* = 7 to 8 mice. Results are from a single experiment.

Fecal metabolomics was used to assess how gut metabolism changed during RSV infection. Mice were intranasally infected with RSV, and fecal samples were taken every day for 7 days. The samples were processed, analyzed by mass spectrometry, and annotated (34, 35). There were 803 detected biochemicals, 705 of which could be identified. The overall fecal metabolomic profile significantly shifted over time following RSV infection (*P* = 0.02) (Fig. 5A). When comparing each time point to baseline, days 3, 6, and 7 had significantly different fecal metabolic compositions, which coincided with when the most significant weight loss occurred (Fig. S6A).

Seventy-six subpathways contained biochemicals which were significantly altered in abundance at day 7 compared to that at day 0 (see Fig. S7). Fifty-three of these pathways had a pathway enrichment value greater than 1, and 23 of these belonged to the lipid metabolism superpathway (Fig. 5B). As seen previously, RSV infection significantly altered the gut microbiota (Fig. S6B to D). To link changes seen in the metabolome with the changes in the gut microbiota, we used PICRUSt to perform a predictive metagenomic analysis. There was a significant increase in the relative abundance of 16S rRNA gene sequences with predicted orthology functions in lipid metabolism at day 7 after RSV infection (*P* < 0.05) (Fig. 5C and D). This reflected the observed change in the fecal metabolome, suggesting that changes in lipid metabolism were driven by the changed gut microbiota.

**FIG 6:**
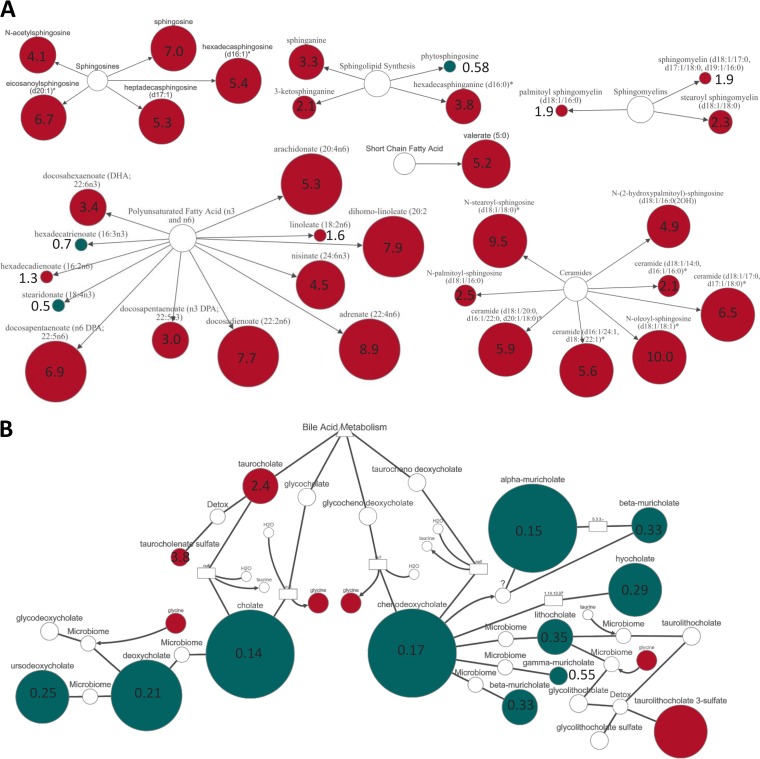
Fold change of individual lipid metabolites in the feces following RSV infection. Metabolites which were significantly altered in abundance from day 0 to day 7 belonged to the sphingosine, sphingolipid, sphingomyelin, ceramide, PUFA \, and SCFA subpathways (A) and to secondary bile acid metabolism (B). Red indicates a significant (*P* ≤ 0.05) increase in the abundance of metabolites at day 7 post-RSV infection (RSV D7/D0, metabolite ratio is ≥1.00). Green indicates a significant (*P* ≤ 0.05) decrease in metabolite abundance (RSV D7/D0 metabolite ratio is <1.00). Size of node indicates magnitude of fold change in abundance but is relative for each pathway. Number inside the node is the exact fold change value.

The sphingolipid and fatty acid metabolism pathways had some of the highest pathway enrichment values following RSV infection. Metabolites within the sphingosines, sphingolipids, sphingomyelins, and ceramide subpathways were significantly increased in abundance at day 7 after RSV infection compared to that at baseline (Fig. 6A). Multiple PUFAs were increased in abundance following RSV infection, including the anti-inflammatory DHA. The SCFA valerate was also significantly increased in abundance following RSV infection (Fig. 6A).

In fecal metabolomics, it can be difficult to determine whether the biochemicals detected are produced via host or microbiota metabolism, because there is often crossover between the sources. However, bile acids can be used to distinguish between host and microbiota metabolism. Primary bile acids are synthesized by the liver, and secondary bile acid metabolism is conducted by gut bacteria. Primary and secondary bile acid metabolism were found to have pathway enrichment values of 1.71 and 1.36, respectively. Several primary bile acids, including cholate and chenodeoxycholate, had decreased abundance at day 7 after RSV infection (Fig. 6B). This corresponded with decreased abundance of the secondary bile acids deoxycholate and lithocholate. Overall, the fecal metabolome was significantly different following RSV infection, with noticeable changes in lipid metabolism.

**FIG 7:**
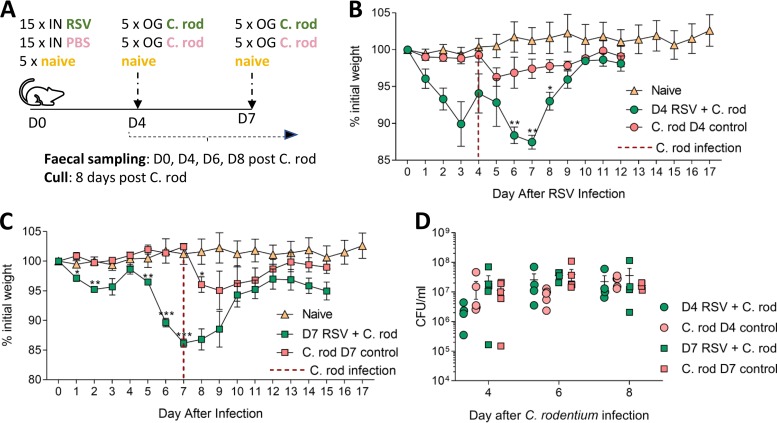
RSV infection does not increase susceptibility to gut infection with Citrobacter rodentium. (A) Mice were intranasally (IN) infected with RSV, intranasally dosed with sterile PBS, or left naive. At day 4 post-RSV infection, one group of infected mice and one group of PBS mice were orally gavaged (OG) with 8 × 10^9^ CFU/ml Citrobacter rodentium (C. rod). This was repeated at day 7 post-RSV infection with separate groups of infected and control mice. Fecal samples were taken before C. rodentium infection and at days 4, 6, and 8 after C. rodentium infection. Mice were culled 8 days after C. rodentium infection. Naive mice were monitored throughout to ensure no C. rodentium contamination occurred. Weights were measured after infection at day 4 (B) or day 7 (C) after C. rodentium infection. (D) C. rodentium CFU/ml in the feces of RSV infected mice. *n* = 4 to 5 mice per group. Results are representative of two experiments. Experiment shown is representative of two independent repeats. Two-way RM ANOVA with Dunnet’s multiple-comparison test. *, *P ≤ *0.05; **, *P ≤ *0.01 ***; *P ≤ *0.001.

To investigate whether respiratory virus infection-associated changes in gut microbiota and fecal metabolome altered colonization resistance and increased susceptibility to gut infection, mice were infected first with RSV and then with the murine enteropathogen Citrobacter rodentium. C. rodentium infection is a model for human enterohemorrhagic Escherichia coli infection and naturally infects all mouse strains, causing mild to moderate disease in BALB/c mice. Mice were split into five groups of five mice per group. Two groups were infected with RSV, two groups were dosed with PBS, and one group was left naive. At day 4 after RSV infection, one RSV group and one PBS group were infected with C. rodentium. At day 7, the remaining RSV and PBS groups were infected with C. rodentium (Fig. 7A). These time points were chosen, as day 3 to day 4 is when the peak viral load and peak initial weight loss occur, while on day 7, there is peak total weight loss, when large changes in the microbiome were observed. Fecal samples were taken from every group before RSV infection and from the naive group at every sampling time point and plated onto C. rodentium selective agar to ensure no cross-contamination had occurred. Fecal samples were taken and analyzed at days 4, 6, and 8 after C. rodentium infection to see if there was any difference in C. rodentium load between the dual- and single-infected groups. Weight loss was not exacerbated when RSV-infected mice were infected with C. rodentium at either day 4 (Fig. 7B) or 7 (Fig. 7C). There was no difference in C. rodentium bacterial load in the feces at days 4, 6, or 8 between dual-infected mice and single-infected mice (Fig. 7D), suggesting that RSV infection did not result in a more permissive or toxic gut environment for C. rodentium growth.

## DISCUSSION

Other studies in mice have observed that respiratory virus infection is associated with a change in the gut microbiota, but none have proposed that infection-induced inappetence is the driver (7–9, 11). Bartley et al. (9) observed that influenza infection drives the gut microbiota composition toward a profile similar to that of calorie-restricted mice. Deriu et al. (8) did not observe any effect on gut microbiota composition when interferon-α/β receptor (IFNAR)^−/−^ mice were infected with influenza virus. This was despite the fact that the mice still lost weight after infection and that IFNAR^−/−^ mice are reported to experience more severe weight loss following respiratory viral infection (36). Yildiz et al. (11) observed that the decrease in gut bacterial load coincided with weight loss after influenza infection, but they did not find any significant correlation between percentage body weight and 16S rRNA gene quantitative PCR (qPCR) data. However, as the authors note, there was a discrepancy between their abundance data when measured via 16S rRNA gene qPCR versus 16S rRNA gene sequencing.

One question remains as to what is driving the alteration in food intake during respiratory viral infection. Our data suggest that weight change is independent of viral load, as after CD8^+^ depletion and TNF-α blockade, lung viral load increased without increasing weight loss, mirroring findings previously reported (26, 28). The strongest effect on weight loss after infection was by depleting CD8^+^ cells (29). Interestingly, the depletion of CD8^+^ cells prevented the reduction in food intake and reversed the changes in the gut microbiota. While CD8 is predominantly present on T cells, it is also found on subsets of dendritic cells and natural killer cells, and we did not explore the effect of CD8^+^ depletion on these cell populations (37, 38). A role for T cells in gut microbiota changes during influenza infection has also been proposed by Wang et al. (7), who linked the changes with IFN-γ production by lung CD4^+^ T cells that had tracked to the gut. While we previously observed RSV-specific CD8^+^ T cells tracking to different tissues, such as the spleen, after infection (39), we did not look in the intestines in the present or previous study. It should be noted that we observed no increase in lymphoid infiltration in the gut after RSV or influenza infection (10). This leads us to think that the effect of CD8^+^ T cells on inappetence is via a secreted factor.

This secreted factor is most likely a cytokine or chemokine produced by CD8^+^ T cells. A number of cytokines have been previously linked to anorexia, including IL-6, TNF-α, CXCL8, IL-1β, IL-18, IL-2, and IFN-γ (40, 41). How these cytokines suppress the normal desire to eat is not well understood, especially in the context of viral infection, as most models that are focused on dissecting the mechanism behind infection-induced inappetence have used lipopolysaccharide (LPS) or peptidoglycan stimulation (13). There is evidence suggesting both direct and indirect effects of these cytokines on the peripheral and central nervous systems, in particular, on the hypothalamus, to suppress the desire to eat (13, 42). Our current study suggests that TNF-α is not the sole factor driving weight loss after viral infection. Likewise, the blockade of IFN-γ had no effect on weight loss after RSV infection. Another contender would be IL-6; however, the blockade of IL-6 during RSV infection has been shown to enhance weight loss (43). Little is known about the role of the other cytokines in inappetence during respiratory viral infection (43). In the present study, we saw reduced levels of IL-5, IL-1α, and IL-1β after CD8 depletion; these might have an impact. Although, the anti-inflammatory drug indomethacin, which has been shown to prevent IL-1β-induced anorexia, did not affect food intake during influenza virus infection (14). Future work should be focused on identifying the link between the induced immune response to infection and changes in sickness behaviors and the gut microbiota.

The biggest changes in the gut metabolome after RSV infection were observed in lipid metabolism. Similar changes were seen in predicted KEGG pathways associated with lipid metabolism in infants with viral bronchiolitis, and an increase in predicted sphingosine gene abundance correlated with increased *Bacteroides* in the gut microbiota (44). This, and the PICRUSt data in our study, suggest that increased sphingosine metabolism following RSV infection may be due to a change in gut microbiota metabolism. Sphingolipid metabolite abundance has also been observed to be altered in the lung metabolome after RSV infection in mice (45) and in the airways of children with RSV bronchiolitis (46). It is interesting to speculate whether altered gut metabolites promote host resistance to the infection or tolerance to the immunopathology (5, 15). Wang et al. (15) used fatal bacterial and viral infections to investigate the benefits of inappetence. They observed that in viral infections, reduced food intake, in particular, glucose, is harmful. We looked further downstream at the effect of infection on the gut microbiota and metabolome, which, due to the coevolved nature of host and microbiota, could also form part of the protective effect of inappetence during infection by altering gut microbiota metabolism. While we do not know the effect of appetite loss on disease outcome, interestingly, a number of proresolving lipids in the guts at the peak of infection were increased. Whether these have an effect on the resolution of infection remains an intriguing question worthy of further investigations. One possibility we investigated was the effect on susceptibility to gut bacterial infections. Previous studies have indicated that influenza infection increased susceptibility to Salmonella enterica serovar Typhimurium (8); however, this is not a natural murine pathogen, and the mice had to be pretreated with streptomycin before infection, which may have masked the impact of the microbiome changes. In our study, we saw no effect of RSV infection on colonization with the mouse enteropathogen Citrobacter rodentium.

The translational impacts of our findings are as yet unclear. The infectious dose used in the mouse model was relatively high compared to a natural infection to ensure a high infection rate: it is of interest that human challenge experiments use a similarly high dose to ensure infection occurs (47). While it is possible that such a high dose leads to a systemic viremia, this is not something we have previously observed, and in other viral lung infection models, we do not observe systemic increases in cytokines outside the lung (48). Overall, in this study, we saw a similar pattern to our previous findings that RSV infection alters the gut microbiome (10) and demonstrate, for the first time, that these alterations are linked to immune-mediated inappetence.

## MATERIALS AND METHODS

### Animals.

Adult female BALB/c (H-2^d^) specific-pathogen-free (SPF) mice were purchased from Charles River Laboratories, UK, and maintained in autoclaved individually ventilated cages (IVC) under positive pressure, with a mixture of Tapvei Eco-Pure Premium Aspen chips (Datesand) and Sizzle-Pet (1034015; LBS, UK) for bedding. Mice were housed in groups of five animals per cage, with the exception of fasting studies, where mice were housed individually to control food intake. Mice had *ad libitum* access to irradiated RM3 pellets for food (801700; SDS, UK), except for the fasting studies, where a set amount of RM3 pellets was provided each day. Food intake was measured during an experiment by weighing the contents of the food holder at the same time every day and dividing the amount of food eaten by the number of mice in the cage. All mice had *ad libitum* access to reverse-osmosis autoclaved water. Feces were collected from individual mice before intervention/infection and during the time course of the experiment. Mice were placed into individual disinfected pots, and one pair of sterile tweezers per mouse was used to collect fecal pellets. Pellets were stored in sterile 1.5-ml Eppendorf tubes at −80°C prior to bacterial DNA extraction. Pots were thoroughly disinfected between mice. At the end of the experiment, mice were culled using intraperitoneal (i.p.) injections with 200 μl pentobarbital, followed by cervical dislocation or exsanguination under terminal anesthesia.

All *in vivo* experiments were performed in the same specific-pathogen-free (SPF) room, which was maintained on a 12-h light/dark cycle at 20 to 24°C with 55% ± 10% humidity, at the Imperial College London, St Mary’s Hospital Campus. Experiments were conducted in accordance with the United Kingdom’s Home Office regulations under protocol number 1. All work was approved by the Animal Welfare and Ethical Review board at Imperial College London, and studies were in accordance with the Animal Research: Reporting of *In Vivo* Experiments (ARRIVE) guidelines.

### Fasting studies.

All forms of enrichment, which could have been eaten during periods of fasting, were removed. Access to food *ad libitum* was also removed, and dry food pellets (RM3; SDS) were cut up, weighed, and placed into glass petri dishes inside the cage. Mice were weighted every day, and 1.3 g of food was placed in the dish every 24 h for a maximum of 3 days or until 15% weight loss was reached, whichever occurred first. Control mice were given 4.3 g food every day. Individually housed mice with *ad libitum* access to food were maintained and sampled alongside fasted/control mice to account for the stress of solo housing.

### Respiratory infections.

Mice were anaesthetized via inhalation of isoflurane and intranasally infected with 100 μl of 2 × 10^6^ PFU/ml RSV, 100 μl 4 × 10^5^ PFU/ml A/Eng/195/2009 influenza virus, or 100 μl PBS per mouse.

### RSV L gene qPCR.

RNA was extracted from lung samples using phenol (QIAzol, 79306; Qiagen) and chloroform extraction and a TissueLyzer (Qiagen, Manchester, UK). RNA was converted into cDNA using a GoScript reverse transcription system (product code A5001; Promega, UK). qPCR for the RSV L gene was performed on a Stratagene Mx 3005p (Agilent technologies, Santa Clara, CA, USA) using the primers 5′-GAACTCAGTGTAGGTAGAATGTTTGCA-3′ and 5′-TTCAGCTATCATTTTCTCTGCCAA-3′ and probe 5′-6-carboxyfluorescein (FAM)-TTTGAACCTGTCTGAACAT-6-carboxytetramethylrhodamine (TAMRA)-3′. RNA copy number per microgram lung RNA was determined using an RSV L gene standard (49).

### TNF-α or IFN-γ depletion.

Mice were i.p. injected with 450 μg monoclonal anti-mouse TNF-α antibody (XT3.11, BE00058; BioXCell), 450 μg monoclonal rat IgG1 isotype control (clone TNP6A7, BE0290; BioXCell), or 500 μg anti-mouse IFN-γ antibody (XMG1.2; BioXCell) 4 h before being intranasally infected with RSV. Mice were i.p. injected again with the same antibodies on days 2, 4, and 6 postinfection.

### Increasing airway TNF-α.

Mice were intranasally dosed with 3 μg in 20 μl per mouse recombinant murine TNF-α (r-TNF-α) (carrier free, 575204; BioLegend Ltd., UK) every day for 3 days. Control mice were intranasally dosed with PBS every day for 3 days.

### CD8^+^ T cell depletion.

Mice were i.p. injected with 500 μg monoclonal anti-mouse CD8α antibody (clone 53-6.72, BE0004-1; BioXCell) or 500 μg monoclonal rat IgG2a isotype control (clone 2A3, BE0089; BioXCell) 24 h before being intranasally infected with RSV. Mice were i.p. injected again with the same antibodies on days 2 and 5 postinfection.

### Flow cytometry.

Bronchoalveolar lavage fluid (BALF) was collected by flushing the lungs three times with 0.5 ml sterile PBS through the trachea. The superior right lung lobe was mashed through a cell strainer and treated with ammonium-chloride-potassium (ACK) lysis buffer (10-5483; Lonza). Cells were pelleted, washed with 1% bovine serum albumin (BSA), 0.2 mM EDTA in PBS, and incubated with Fixable Live/Dead Aqua fluorescent reactive dye (L34966; Invitrogen), anti-mouse CD16/CD32 (Fc block, clone 2.4G2, 70-0161-V100; Tondo Biosciences), anti-mouse CD3e fluorescein isothiocyanate (FITC) (clone 145-2011, 11-0031-85; eBioscience), anti-mouse CD4 phycoerythrin (PE)/Cy7 (clone GK1.5, 100422; BioLegend), anti-mouse CD8a allophycocyanin (APC)/H7 (clone 53-6.7, 560182; BD Biosciences). Cells were acquired on a BD Fortessa flow cytometer and gated on live CD3^+^ lymphocytes. Data were analyzed on FlowJo v10.1.

### Airway cytokine measurement.

Cytokines in the airways were measured by enzyme-linked immunosorbent assay (ELISA; Bio-Techne) or a Magnetic Luminex assay (R&D Systems).

### 16S rRNA gene sequencing.

Bacterial DNA was extracted from 30 mg feces/mouse/time point using the FastDNA Spin kit for soil (116560200; MP Biomedicals). A control extraction with no sample was performed for each kit and sequenced to monitor bacterial DNA contamination within the kit components. Each sequencing run contained a negative control (nuclease-free water used for library preparation), a positive control (mock community), and a kit control, which corresponded to the kit used to extract DNA from samples. The V4 variable region of the 16S rRNA gene was amplified using universal bacterial primers (50) which were uniquely barcoded for each sample (Illumina Nextera Indexes version 2). The amplicons were purified, quantified, and equimolar pooled to produce a 16S rRNA gene library as described previously (10). Paired-end sequencing of the 8 pM denatured library, spiked with 8 pM of PhiX, was performed using the Illumina MiSeq platform (51).

16S rRNA gene sequencing data were processed using the QIIME 1.9.0 software suite (52) as outlined fully by Groves et al. (10). For microbiota composition analysis, operational taxonomic units (OTUs) were clustered at 97% sequence identify using UCLUST (53) and open reference clustering. Representative OTUs were picked using the SILVA 115 rRNA database. Taxonomy was assigned using the RDP classifier (54) and the SILVA 115 rRNA database for reference sequences. Diversity and phylogenetic analyses were conducted in R 3.3.0 (55) with RStudio (56) using the phyloseq (57) and vegan packages (58). Beta diversity was analyzed using nonmetric multidimensional scaling (NMDS) ordination on a Bray-Curtis dissimilarity matrix.

### PICRUSt.

PICRUSt (Phylogenetic Investigation of Communities by Reconstruction of Unobserved States) predicts the metagenome of a bacterial community using 16S rRNA sequencing data by constructing a table of expected gene abundances for each OTU based on KEGG orthology (59). The 16S rRNA gene sequencing data were analyzed as described above and by Groves et al. (10), with the exception that PICRUSt requires a closed-reference OTU table where representative sequences are picked and taxonomy assigned using an adapted Greengenes database for PICRUSt (60). Consequently, a new OTU table was generated for this. PICRUSt data were analyzed in STAMP (Statistical Analysis of Metagenomes Profiles).

### Fecal metabolomics.

Profiling of the fecal metabolome before and during RSV infection was performed by Metabolon (Durham, NC, USA). Samples were processed, analyzed, and annotated by Metabolon as described previously (34, 35) using ultrahigh-performance liquid chromatography–tandem mass spectroscopy (UPLC-MS/MS).

Analysis was performed on scaled data, where for each metabolite/biochemical, the values were scaled to set the medium equal to one. Any missing values were inputted with the minimum amount for that biochemical. Pathway enrichment analysis was used to determine which metabolic pathways contained significantly more differentially abundant metabolites following RSV infection. To calculate the pathway enrichment value for a metabolic pathway between two time points, the number of metabolites belonging to a particular pathway which were significantly altered in abundance in a pairwise comparison (k) (relative to the overall number of detected metabolites in that specific pathway [m]) were compared to the total number of significantly altered metabolites in that pairwise comparison (n), relative to all detected metabolites in the study (N). Pathway enrichment = (k/m)/(n/N). Metabolic networks and pathways were visualized and fold change in metabolite abundance between time points calculated using Cytoscape software (61).

### Statistics.

All statistical analyses were performed in Graph Pad Prism V6/8, except for the permutational multivariate analysis of variance (PERMANOVA) for differences in beta diversity, which was performed in R version 3.5.0, and statistical analysis of fecal metabolites, which was calculated in Cytoscape or by Metabolon. For weight loss, a repeated measures (RM) two-way analysis of variance (ANOVA) comparing weight at day 0 with weight at each time point was conducted for each group with Dunnett’s multiple-comparison test. Differences in phyla and family abundance between time points and between groups were calculated using RM two-way ANOVA with Sidak’s multiple-comparison test. TNF-α levels, cell counts, and percentages were tested using an ordinary one-way ANOVA, comparing the mean from each group with the mean from every other group and using Dunnett’s multiple-comparison test for correction.

### Data availability.

Sequencing data were deposited in the European Nucleotide Archive under accession number PRJEB32774. Metadata, mapping files, OTU tables, phylogenetic trees, and codes used for analysis were uploaded to BioStudies at EMBL-EBI.

10.1128/mBio.03236-19.6 FIG S6 The gut microbiota is altered during RSV infection: samples matched to those used for metabolomics. Mice infected with RSV. (A) Weight change measured after infection. (B) Gut microbiota diversity measured at days 3 and 7 after infection (C). Relative abundances of *Bacteroidetes* and *Firmicutes* after infection. (D) Family-level gut microbiota analysis after infection. Download FIG S6, TIF file, 1.4 MB. Copyright © 2020 Groves et al. 2020 Groves et al. This content is distributed under the terms of the Creative Commons Attribution 4.0 International license.

10.1128/mBio.03236-19.7 FIG S7 Pathway enrichment analysis of metabolite abundance comparing before infection to day 7. Pathway enrichment analysis comparing the fecal metabolome of day 7 post-RSV infection samples to before infection (day 0). Pathway enrichment values for each subpathway (*x* axis) were calculated by taking the number of significantly altered metabolites in that pathway at day 7 (k), relative to the total number of metabolites detected in that pathway (m), and dividing this by the total number of significantly altered metabolites at day 7 (n), relative to the number of detected metabolites in the study (N). Pathway enrichment = (k/m)/(n/N). Red line indicates pathway enrichment value above and below 1. Download FIG S7, TIF file, 2.1 MB. Copyright © 2020 Groves et al. 2020 Groves et al. This content is distributed under the terms of the Creative Commons Attribution 4.0 International license.
